# A seed expansion-based method to identify essential proteins by integrating protein–protein interaction sub-networks and multiple biological characteristics

**DOI:** 10.1186/s12859-023-05583-8

**Published:** 2023-11-30

**Authors:** He Zhao, Guixia Liu, Xintian Cao

**Affiliations:** 1 College of Computer Science and Technology, Jilin University, Changchun, China; 2 Key Laboratory of Symbolic Computation and Knowledge Engineering of Ministry of Education, Jilin University, Changchun, China

**Keywords:** Essential proteins, PPI sub-networks, Seed expand, Biological data

## Abstract

**Background:**

The identification of essential proteins is of great significance in biology and pathology. However, protein–protein interaction (PPI) data obtained through high-throughput technology include a high number of false positives. To overcome this limitation, numerous computational algorithms based on biological characteristics and topological features have been proposed to identify essential proteins.

**Results:**

In this paper, we propose a novel method named SESN for identifying essential proteins. It is a seed expansion method based on PPI sub-networks and multiple biological characteristics. Firstly, SESN utilizes gene expression data to construct PPI sub-networks. Secondly, seed expansion is performed simultaneously in each sub-network, and the expansion process is based on the topological features of predicted essential proteins. Thirdly, the error correction mechanism is based on multiple biological characteristics and the entire PPI network. Finally, SESN analyzes the impact of each biological characteristic, including protein complex, gene expression data, GO annotations, and subcellular localization, and adopts the biological data with the best experimental results. The output of SESN is a set of predicted essential proteins.

**Conclusions:**

The analysis of each component of SESN indicates the effectiveness of all components. We conduct comparison experiments using three datasets from two species, and the experimental results demonstrate that SESN achieves superior performance compared to other methods.

## Background

Essential proteins are crucial and indispensable for cellular activities [1]. The identification of essential proteins promotes an understanding of the minimal requirements for cell survival and reproduction. The study of essential proteins is beneficial for discovering pathogenic genes and generating novel approaches for disease treatment. [2, 3]. The identification of essential proteins plays a crucial role in advancing research and development in the fields of biology and pathology.

Experimental methods for identifying essential proteins include the following forms: single gene knockouts [4], gene knockdown [5], and RNA interference [6]. Although these methods have high accuracy, the experiment is expensive, time-consuming, inefficient, and there are still species limitations. With the rapid development of bioinformatics, a large amount of PPI data is measured through high-throughput technology. This provides conditions for research at the PPI network level. Research based on PPI networks has become a focal point in the field of bioinformatics [7]. However, PPI data obtained through high-throughput technology include a high rate of false positives [8, 9]. To overcome the impact of this rate, researchers have attempted various methods to construct weighted PPI networks to remove false positive interactions. These network-based methods have been proved to be effective in the identification of essential proteins [10].

Some researchers concentrate on the identification of essential proteins based on the topology of the PPI network. Topology-based methods can generally be divided into three categories: local topology-based, global topology-based and multi-topology-based. Local topology-based methods assess a protein’s essentiality through its local neighborhood, such as Degree Centrality (DC) [11], Eigenvector Centrality (EC) [12], Local Average Connectivity (LAC) [13], and Neighborhood Centrality (NC) [14]. Global topology-based methods, including Betweenness Centrality (BC) [15], Closeness Centrality (CC) [16], Information Centrality (IC) [17], and Subgraph Centrality (SC) [18], measure topological properties globally based on characteristics of paths or shortest paths between proteins. All the above mentioned approaches are included in CytoNCA [19], which is a plugin of Cytoscape. Multi-topology-based methods combine various topological characteristics. For example, SIGEP [20] presents a *p* value calculation method, which utilizes network topology characteristics (degree and local clustering coefficient) as test statistics and can outperform the aforementioned methods. Nonetheless, all these topology-based methods ignore the topology characteristics of predicted essential proteins.

Predicting essential proteins only by using network topology ignores the biological properties of proteins. In recent years, researchers have discovered that the biological characteristics of proteins are closely related to their essentiality. PeC [21] and JDC [22] are developed to identify essential proteins by integrating PPI networks and gene expression data. LNSPF [23] is proposed to identify essential proteins based on gene expression data, subcellular localization, homologous information and topological features. RSG [24] designs essential proteins prediction method based on RNA-Seq, subcellular localization, and GO annotation datasets, the experimental results include two species (Saccharomyces cerevisiae and Drosophila melanogaster). RWEP [25] adopts a random walk algorithm and integrates topological and biological properties to determine protein essentiality in PPI networks, RWEP outperforms PeC and RSG in predicting essential proteins. It incorporates multiple biological properties to enhance the efficiency of essential protein prediction. However, it is unclear which biological data is the most effective. CPPK and CEPPK [26] predict essential proteins by integrating network topology, gene expression data, and certain essential proteins as prior knowledge. However, the performance of CPPK excessively depends on the number of essential proteins. NCCO [27] combines orthology datasets from species S.cerevisiae and E.coli with network topology to predict essential proteins. RWO [28] utilizes orthologous relationships between yeast and human PPI networks. All these methods that integrate PPI network topology with biological data are more effective than those based solely on network topology. However, the specific impact of each biological data and individual components of these methods on the final prediction results remains unknown. Researchers have also proposed some deep learning frameworks that integrate biological features and network topology features to identify esssential proteins. DeepEP [29] utilizes multi-scale convolutional neural networks to extract biological features from gene expression profiles, while the node2vec [30] technique is applied to automatically learn topological features from PPI networks. These features are then concatenated to predict essential proteins. Zeng et al. [31] propose a deep learning framework for automatically learning biological features without prior knowledge. They employ the node2vec technique to automatically acquire a richer representation of the PPI network topology. Bidirectional long short term memory cells [32] are employed to capture non-local relationships in gene expression data. Additionally, they utilize a high-dimensional indicator vector to characterize biological features related to subcellular localization. Yue et al. [33] propose a deep learning framework for predicting essential proteins by integrating features obtained from the PPI network, subcellular localization, and gene expression profiles.

In recent years, some studies have been dedicated to constructing PPI sub-networks through gene expression analysis in order to infer the activity of protein interactions. TS-PIN [34] constructs a network by using gene expression data and subcellular localization to identify essential proteins. TP-WDPIN [35] mines protein complexes from weighted dynamic PPI sub-networks constructed by gene expression data. Inspired by this, we construct PPI sub-networks by gene expression data and perform the process of seed expansion in these sub-networks.

In this research, we propose an effective method for identifying essential proteins, called SESN. SESN is a seed expansion method based on PPI sub-networks and biological characteristics. The PPI network forms an undirected graph, where proteins serve as nodes and protein-protein interactions as edges. To filter false positive interactions in PPI network, we integrate multiple biological characteristics to weight the edges and nodes of the PPI network and construct PPI sub-networks based on gene expression data. Seed expansion is performed simultaneously in each sub-network, and the expansion results of all the sub-networks will be summarized to the whole PPI network. To avoid relying solely on essential proteins, we will not select seeds from the essential proteins dataset. Instead, each sub-network will randomly select a protein as a seed. The expansion process is based on the topological features of the predicted essential proteins in each sub-network. In this process, we select the protein that is most closely related to the predicted essential proteins and add it to the set of predicted essential proteins. The error correction mechanism filters out proteins that have been expanded but exhibit low essentiality. The weight of a protein in the whole PPI network represents the essentiality of this protein. To ensure the ongoing expansion of the set of predicted essential proteins, after removing a protein, we expand the protein with the highest weight that is strongly associated with the predicted essential proteins. SESN evaluates the influence of biological data on experimental outcomes and identifies the most effective data to achieve optimal results. The output of SESN consists of a set of predicted essential proteins expanded by the seeds of all sub-networks. Proteins that expand earlier are given higher rankings. Comparative experiments are conducted across three datasets from two species. The experimental results demonstrate that, when compared with other methods(CPPK, CEPPK, RWEP, SIGEP, RWO and TS-PIN), SESN achieves the best results across three datasets. Analysis of each component within SESN reveals that all components are effective, with particular emphasis on the error correction mechanism.

The contributions of SESN are outlined as follows: (1) SESN constructs weighted PPI sub-networks by integrating multiple biological data and conducts simultaneous seed expansion within each sub-network. (2) The seed expansion process integrates the topological characteristics of predicted essential proteins in the sub-networks. (3) The error correction mechanism integrates the topological characteristics of predicted essential proteins in the whole PPI network. (4) SESN selects the biological data yielding the best experimental results and integrates multiple biological characteristics to assign weights to both PPI sub-networks and the whole PPI network.

**Fig. 1:**
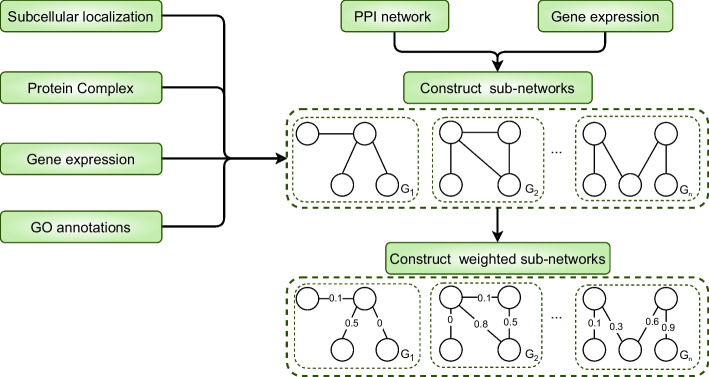
The process of constructing weighted sub-networks

**Fig. 2:**
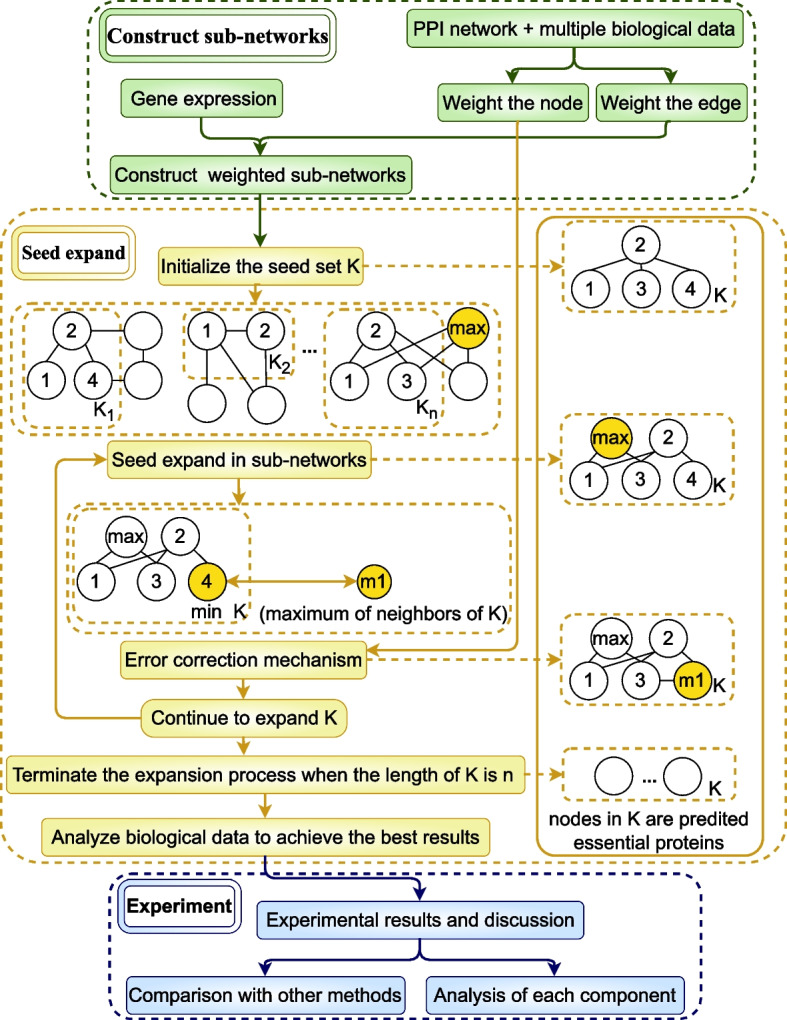
The overall process of SESN

The overall process of SESN is shown in Fig. 2, which provides an example to illustrate the process of seed expansion. The green part represents the initialization of the weighted PPI network and the weighed sub-networks. The detailed process of constructing weighted sub-networks is shown in Fig. 1. The yellow part represents the seed expansion process, and the yellow rounded rectangle on the right illustrates the expansion process of *K*(the definition of *K* is provided in section ’Initialize the seed set and the weight of node or edge’). Initially, there are 4 sub-networks, so *K* is initialized with 4 nodes named node 1, 2, 3 and 4. Then, the expansion process is performed simultaneously in these 4 sub-networks. The node with the highest weight is chosen and added to *K*. Following this, the error correction mechanism filters out the node with the lowest weight in *K* and introduces node *m*1. Among all the neighbors of *K*, *m*1 is the node with the largest weight and the closest connection to *K*. The expansion of *K* persists until its length reaches the output length *n*. The blue part illustrates the experimental process of SESN, to prove the superiority and effectiveness of SESN, we compare SESN with other methods and analyze each component of SESN.

## Methods

### Experimental datasets

To prove the superiority of SESN across different species, experiments are conducted on Saccharomyces cerevisiae and Drosophila melanogaster. We utilize PPI datasets, essential proteins, protein complexes, gene expression data, GO annotations and subcellular localization. Additionally, we perform ID mapping across different datasets using UniProt(https://www.uniprot.org/) as a reference.

**PPI datasets** For Saccharomyces cerevisiae, the PPI dataset can be downloaded from DIP [36](version of 20101010), and BioGRID [37]. As for Drosophila melanogaster, the PPI dataset is download from BioGRID, to distinguish it from Saccharomyces cerevisiae, it is denoted as fruitfly. The number of proteins and essential proteins and other relevant information for these datasets are presented in Table 1.

**Table 1:** Information of PPI datasets

*Datasets*	*Proteins*	*Interations*	*Essentialproteins*
DIP	5093	24,743	1167
BioGRID	5640	59,748	1200
Fruitfly	7783	35,015	493

**Essential proteins** For Saccharomyces cerevisiae, essential proteins are selected from MIPS [38], SGD [39], DEG [40], and OGEE [41], and there are 1285 essential proteins in total. In the case of Drosophila melanogaster, essential proteins are selected from DEG and OGEE, after ID mapping and the removal of duplicate proteins, the fruitfly PPI dataset contains 493 essential proteins.

**Protein complex** For Saccharomyces cerevisiae, protein complexes are collected from MIPS, SGD, ALOY [42], and CYC2008 [43, 44]. Only protein complexes containing two or more proteins are retained, resulting in a total of 745 protein complexes in the final dataset. For Drosophila melanogaster, protein complexes are obtained from AP-MS [45]. After mapping these complexes with the fruitfly PPI datasets, the dataset encompasses 1637 protein complexes.

**Gene expression data** Gene expression data of Saccharomyces cerevisiae and Drosophila melanogaster can be downloaded from GEO (https://www.ncbi.nlm.nih.gov/geo/browse/) with accession GSE3431 [46] and GSE7763 [47], respectively. The probe data matrix for Saccharomyces cerevisiae consists of 9335 rows, while the probe data matrix for Drosophila melanogaster comprises 18952 rows. To map with PPI datasets, we download SOFT formatted family files from GEO. In cases where multiple probe data correspond to a single ID of PPI datasets, we take the average value of multiple probe data. After preprocessing, we obtain 4981, 5318, and 7378 gene expression data for DIP, BioGRID, and fruitfly, respectively.

**GO annotations and Subcellular localization** For Saccharomyces cerevisiae, GO annotation data is available from(https://downloads.yeastgenome.org/curation/literature/go_slim_mapping.tab). For Drosophila melanogaster, GO annotation data is extracted from the COMPARTMENTS database [48]. Subcellular localization is downloaded from the knowledge channel of the COMPARTMENTS database.

### Gene expression data-based method for constructing PPI sub-networks

Gene expression data is presented in the form of an expression matrix, where each row represents the expression level of a protein, and each column corresponds to the expression level of a sample point. The number of sample points varies across different species. In the case of Saccharomyces cerevisiae, there are 12 sample points, while for Drosophila melanogaster, there are 34 sample points. Each sample point corresponds to an average gene expression value.

For gene *g* of Saccharomyces cerevisiae, the average gene expression value can be expressed as Eq. 1.

1 $$\begin{aligned} Ge_{i}(g)=\frac{expr_{i}(g) + expr_{i+12}(g) + expr_{i+24}(g)}{3}, i \in [0,11] \end{aligned}$$

where $expr_{i}(g)$ represents the gene expression value from the expression matrix, and *i* denotes the sample point number. The gene expression data of Saccharomyces cerevisiae comprises three cell cycles, each containing 12 time points. Each sample point corresponds to the average gene expression value at a specific time point across the three cell cycles. For gene *g* of Drosophila melanogaster, the average gene expression value can be expressed as Eq. 2.

2 $$\begin{aligned} Ge_{i}(g)=\frac{expr_{4 \times i}(g) + expr_{4 \times i +1}(g) + expr_{4 \times i +2}(g) + expr_{4 \times i +3}(g)}{4}, i \in [0,33] \end{aligned}$$

where $expr_{i}(g)$ represents the gene expression value in the expression matrix, and *i* signifies the sample point number. The gene expression data for Drosophila melanogaster consists of 136 column values, with every set of 4 columns corresponding to 4 repeated experiments for one sample.

Based on gene expression data, we can construct PPI sub-networks. Saccharomyces cerevisiae contains 12 sub-networks, and Drosophila melanogaster contains 34 sub-networks. PPI networks can be abstracted into graph $G=(V,E)$, where *V* is a set of nodes, *E* is a set of edges. Proteins are abstracted into nodes, and protein-protein interactions are abstracted into edges. Sub-networks based on gene expression data can be represented as $G_{i}$, *G* can be represented as $G_{i}$, forming $G=\{G_{1},G_{2},\dots ,G_{i},\dots ,G_{n}\}$, where *n* represents the number of sample points of gene expression data. Each $G_{i}=(V_{i},E_{i})$ is a sub-network of *G*, with $V_{i} \subseteq V$, $E_{i} \subseteq E$. For any edge $e \in E_{i}$, the protein pairs in *e* are denoted as $v_{a}$ and $v_{b}$. Only if both $v_{a}$ and $v_{b}$ are actively expressed at sample point *i*, will *e* be added to $E_{i}$. This approach effectively filters out noisy edges from sub-network $G_{i}$.

If the gene expression value of the sample point is greater than the threshold, the corresponding protein is considered to be active at this sample point. So, how to determine the threshold? The 3-sigma model calculates the active expression threshold of each protein according to the characteristics of the expression value curve [49]. For gene *g*, the arithmetic mean and standard deviation of its gene expression data are *Avg*(*g*) and $\sigma (g)$, respectively. *Avg*(*g*) and $\sigma (g)$ can be expressed as follows:

3 $$\begin{aligned} Avg(g)= & {} \frac{\sum _{i=1}^{n} Ge_{i}(g)}{n}, \end{aligned}$$

4 $$\begin{aligned} \sigma (g)= & {} \sqrt{\frac{\sum _{i=1}^{n}\left( Ge_{i}(g)-Avg(g)\right) ^{2}}{n-1}}. \end{aligned}$$

where *n* is the number of sample points of gene expression data. The value of $\sigma (g)$ reflects the fluctuation of gene expression data. *k*-sigma (*k*=1,2,3) threshold is calculated by three-sigma method [50–53], which is defined as Eq. 5.

5 $$\begin{aligned} Thr_{k}(g)=Avg(g)+k \cdot \sigma (g) \cdot \left( 1-\frac{1}{1+\sigma ^{2}(g)}\right) , \end{aligned}$$

where *Avg*(*g*) and $\sigma (g)$ are calculated by Eqs. 3 and  4, respectively. If $\sigma (g)$ is very small, $Ge_{i}(g)$ is close to *Avg*(*g*), and $Thr_{k}(g)$ is close to *Avg*(*g*). Conversely, if $\sigma (g)$ is very large, $Ge_{i}(g)$ is not concentrated around *Avg*(*g*), but represents a set of strongly oscillating data. In such cases, $Thr_{k}(g)$ is close to $Avg(g)+k \cdot \sigma (g)$, where *k* is a multiple of $\sigma (g)$, $Thr_{k}(g)$ is positively correlated with *k*, a larger *k* results in a higher $Thr_{k}(g)$. When $k=3$, $Thr_{k}(g)$ achieves the highest confidence. For instance, if $Ge_{i}(g)\ge Thr_{3}(g)$, $Ap_{i}(g)$ get the largest value 0.99(as defined by Eq. 6).

It is assumed that a set of gene expression data follows a probability distribution similar to the normal distribution. If this assumption is correct, the mean and variance of this group of data are denoted as $\mu$ and $\sigma$, respectively, then, $\ \textrm{P}\{|x-\mu |<3 \sigma \} \approx 0.99,\ \textrm{P}\{|x-\mu |<2 \sigma \} \approx 0.95$, and$\ \textrm{P}\{|x-\mu |< \sigma \} \approx 0.68$. Based on this theory, the probability of active expression of gene *g* at sample point *i* can be calculated as follows:

6 $$\begin{aligned} \begin{aligned} Ap_{i}(g)=\left\{ \begin{array}{lcr}{0.99}\quad if\ Ge_{i}(g)\ge Thr_{3}(g)\\ {0.95}\quad if\ Thr_{3}(g)> Ge_{i}(g)\ge Thr_{2}(g)\\ {0.68}\quad if\ Thr_{2}(g) > Ge_{i}(g)\ge Thr_{1}(g)\\ {0.0}\quad \; if\ Ge_{i}(g) < Thr_{1}(g)\\ \end{array}\right. \end{aligned} \end{aligned}$$

To further measure the reliability of protein interaction edges in each sub-network, we construct weighted sub-graphs. For an edge $e=(v,u) \in E_{i}$ in the weighted sub-graph $G_{i}=(V_{i},E_{i},W_{i})$, where protein *v* corresponding to gene *v* and protein *u* corresponding to gene *u*, we define $W_{i}(v,u)=Ap_{i}(v) \cdot Ap_{i}(u)$, the weight of an edge represents the possibility that both gene *v* and gene *u* are active. ID mapping of gene and protein has been done in section ’Experimental datasets’.

### Biological data-based method for weighting proteins and protein-protein interactions.

The essentiality of protein is associated with some biological data, such as protein complex, gene expression data, GO annotations and subcellular localization. We utilize multiple types of biological data to characterize the essentiality of protein-protein interactions.

#### GO annotations

GO terms annotate the functional properties of a protein. For two interacted proteins, the more common GO terms they have, the more similar their functions are, and the greater weight of their interaction is [35]. The weight of an edge based on GO annotations is denoted as Eq. 7.

7 $$\begin{aligned} GOW(v,u) = \frac{|GO_{v} \cap GO_{u} |^2}{|GO_{v} |\cdot |GO_{u} |} \end{aligned}$$

where $GO_{v}$ is the set of GO terms of protein *v*. We use *GOW*(*v*, *u*) to assign the weight of the edge (*v*, *u*).

#### Gene expression

The interaction between two proteins can be weighted based on the strength of their co-expression, as demonstrated in previous studies [21]. The weight is determined by the Pearson correlation coefficient (PCC) calculated from gene expression data [21, 54]. PCC is denoted as follows:

8 $$\begin{aligned} PCC(X,Y) = \frac{\sum _{k=1}^{n} (X_{k}-\bar{X})(Y_{k}-\bar{Y})}{\sqrt{\sum _{k=1}^{n} (X_{k}-\bar{X})^2}\sqrt{\sum _{k=1}^{n} (Y_{k}-\bar{Y})^2}} \end{aligned}$$

where *X* and *Y* correspond to the gene expression data of protein *v* and protein *u* respectively. $X=\{X_{1},X_{2},\dots ,X_{i},\dots ,X_{n}\}$, $Y=\{Y_{1},Y_{2},\dots ,Y_{i},\dots ,Y_{n}\}$, *n* is the number of sample points of gene expression data, which is defined in section ’Construct PPI sub-networks by gene expression data’. $X_{i} = Ge_{i}(v)$, and $Y_{i} = Ge_{i}(u)$, gene *v* and gene *u* correspond to protein *v* and protein *u*, for Saccharomyces cerevisiae, $X_{i}$ and $Y_{i}$ are defined by Eq. 1, and for Drosophila melanogaster, $X_{i}$ and $Y_{i}$ are defined by Eq. 2.

Since PCC ranges from $[-1,+1]$, it needs to be standardized. *GW*(*v*, *u*) is the standardization of PCC, which is denoted as Eq. 9.

9 $$\begin{aligned} GW(v,u) = \frac{PCC(X,Y)+1}{2} \end{aligned}$$

where *PCC*(*X*, *Y*) is denoted as Eq. 8. *GW*(*v*, *u*) ranges from $[0,+1]$. We finally use *GW*(*v*, *u*) to calculate the weight of the edge (*v*, *u*).

#### Protein complex

Proteins typically carry out biological functions through participation in protein complexes. A protein’s likelihood of being essential often increases with the number of protein complexes it is involved in, as highlighted in previous studies [55, 56]. Consequently, the count of protein complexes in which a protein is located can reflect its essentiality. $PC_{v}$ denotes the number of protein complexes in which the protein *v* is located. Additionally, $PC_{max} = max\left( PC_{v}\right) , (v \in V)$. The weight of edge (*v*, *u*) is denoted as Eq. 10.

10 $$\begin{aligned} PCW(v,u) = \frac{PC_{v} \cdot PC_{u}}{PC_{max}^{2}} \end{aligned}$$

#### Subcellular localization

The essentiality of proteins is related to their subcellular localizations, some subcellular localizations have a strong correlation with the essentiality of protein [57, 58]. In this section, we firstly select some subcellular localizations which are more relevant to essential proteins, and then, these selected subcellular localizations are weighted according to how important they are. Lastly, we score proteins’ essentiality by the subcellular localizations they appeared.

**Fig. 3:**
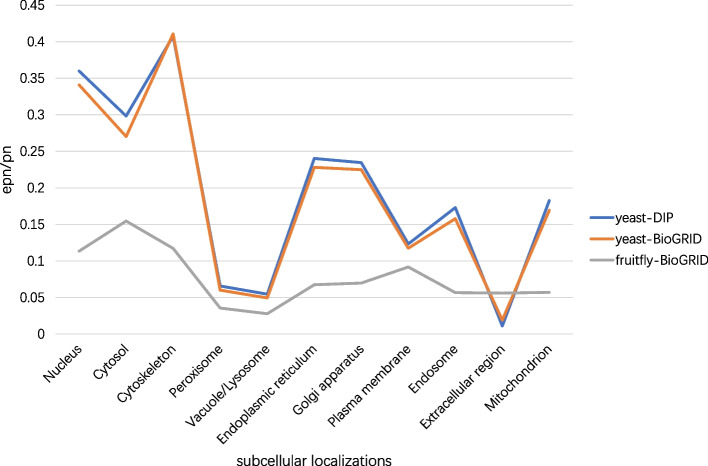
Among 11 subcellular localizations, *epn*/*pn* is the proportion *EPI*, which is denoted as Eq. 11. Nucleus, Cytosol, Cytoskeleton, Peroxisome, Vacuole/Lysosome, Endoplasmic reticulum, Golgi apparatus, Plasma membrane, Endosome, Extracellular region, and Mitochondrion are 11 subcellular localizations, respectively. Especially, in datasets DIP and BioGRID, subcellular localization Vacuole/Lysosome is Vacuole. In the dataset fruitfly, it is referred to as Lysosome

Subcellular localizations usually contain 11 compartments [48]. For the 11 subcellular localizations, we calculate the proportion *EPI* as follows:

11 $$\begin{aligned} EPI_{subi} = \frac{EP_{subi}}{P_{subi}}, subi \in [1,11] \end{aligned}$$

where *subi* is the count of the 11 subcellular localizations, $EP_{subi}$ is the number of essential proteins in *subi*, and $P_{subi}$ is the number of proteins in *subi*. For the 11 subcellular localizations of different datasets, the proportion *EPI* is shown in Fig. 3.

As shown in Fig. 3, the proportion *EPI* of some subcellular localizations is significantly higher than others, such as Nucleus, Cytosol, and Cytoskeleton. On the contrary, the proportion *EPI* of Peroxisome and Extracellular region is significantly lower than others. This characteristic is present in different species, such as saccharomyces cerevisiae and drosophila melanogaster. Therefore, we select subcellular localizations we used based on the DIP dataset. The threshold *EPthre* is denoted as Eq. 12.

12 $$\begin{aligned} EPthre = \frac{ep}{p} \end{aligned}$$

where, *ep* is the number of essential proteins in datasets DIP, and *p* is the number of proteins in datasets DIP. If *EPI* of subcellular localization is greater than threshold *EPthre*, this subcellular localization is selected in set *SC*. $SC = \{Nucleus, Cytosol, Cytoskeleton, Endoplasmic reticulum, Golgi apparatus\}$. For every selected subcellular localization $SC_{i}$, we score it by the number of proteins in it, and it is denoted as Eq. 13.

13 $$\begin{aligned} SCS_{i} = \frac{NSC_{i}}{NSC_{max}} \end{aligned}$$

where, $NSC_{i} = number \; of \; proteins \; in \; SC_{i}$, $NSC_{max} = max(NSC_{i})$, $SCS_{i}$ ranges from $[0,+1]$. Protein *v* is weighted based on the subcellular localization score, and its weight is denoted by Eq. 14.

14 $$\begin{aligned} SW_{v} = \frac{SSC_{v}}{SSC_{max}} \end{aligned}$$

where, $SSC_{v}=\sum \limits _{v \in SC_{i}} SCS_{i}$, $SSC_{max} = max\left( SSC_{v}\right) , (v \in V)$. $SW_{v}$ ranges from $[0,+1]$

### Seed expansion method based on sub-networks and biological data

Essential proteins have a close relationship with each other [26]. CPPK predicts essential proteins by integrating network topology and some essential proteins as prior knowledge. However, the performance of CPPK depends excessively on the number of essential proteins used as prior knowledge. To tackle this problem, we randomly select a protein as a seed in each sub-network, and the expansion process is based on the seed set of each sub-networks. This integrates the topological characteristics of predicted essential proteins in sub-networks. The error correction mechanism filters out proteins that have been expanded but are of low essentiality. This mechanism integrates the topological characteristics of predicted essential proteins in the whole PPI network, and the weight of each node is based on biological data. To provide a more realistic representation of protein interactions, we divide the protein-protein interaction network into several sub-networks based on gene expression data. In this section, we execute seed expansion in each sub-network simultaneously and summarize the expansion results to the whole PPI network. The detailed proecess of seed expansion is presented in Algorithm 1.

**Algorithm:**
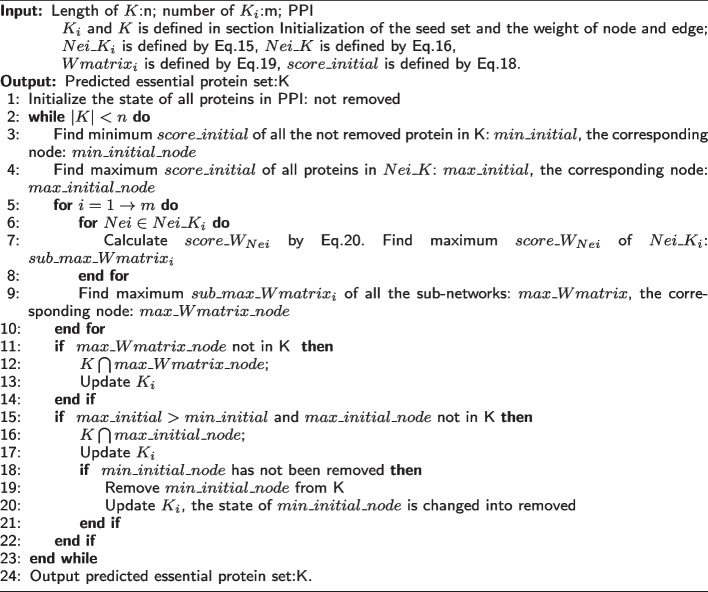
1

#### Initialization of the seed set and the weight of node and edge

For the whole PPI network, the set of predicted essential protein is initialized as *K*. For the PPI sub-networks, the sets of predicted essential protein are initialized as $K_i$, where $i\in [1,m]$, and *m* is the number of sub-networks. A protein is randomly chosen from each sub-network and added to $K_i$, ensuring that the intersection of all initial $K_i$ is empty. The initial *K* is formed by taking the union of all the initial $K_i$ sets, expressed as $K = \bigcup _{i=1}^m K_i$. *K* is the set of predicted essential proteins, and it continues expanding until its length reaches the output length *n*. In other words, the length of output *K* is *n*. The value of *n* is initialized as $\frac{|V|}{4}$ for Saccharomyces cerevisiae and $\frac{|V|}{10}$ for Drosophila melanogaster. When $|K |= n$, the proteins within *K* constitute all the essential proteins predicted by SESN. Furthermore, the ranking of a protein within *K* is higher if it was added earlier.

$Nei\_K_i$ is the union of neighbor sets of all proteins in $K_i$. Subsequently, any protein within $Nei\_K_i$ that is also present in *K* is removed.

15 $$\begin{aligned} Nei\_K_i = (\bigcup _{u \in K_i}^{K_i} N_{u}) \setminus K \end{aligned}$$

where, $N_{u}$ is the set of neighbors of protein *u*. To clarify, $Nei\_K_i \cap K = \emptyset$, indicating that the intersection of $Nei\_K_i$ and *K* is empty. Similarly, $Nei\_K$ is formed by combining the neighbor sets of all proteins in *K*. Subsequently, any protein within $Nei\_K$ that is also present in *K* is removed.

16 $$\begin{aligned} Nei\_K = (\bigcup _{u \in K}^{K} N_{u}) \setminus K \end{aligned}$$

$score\_initial$ describes the essentiality of protein in the whole PPI network. We initialize a weight matrix, defined as Eq. 17.

17 $$\begin{aligned} W_{v,u} = GOW(v,u) \cdot GW(v,u) \cdot PCW(v,u) \end{aligned}$$

where, *GOW*(*v*, *u*) is denoted as Eq. 7, *GW*(*v*, *u*) is denoted as Eq. 9, and *PCW*(*v*, *u*) is denoted as Eq. 10. $W_{v,u}$ is the weight of edge (*v*, *u*), then, we initialize the weight of protein by Eq. 18.

18 $$\begin{aligned} score\_initial_{v} = (\sum _{u \in N_{v}}^{N_{v}}W_{v,u}) \cdot SW_{v} \end{aligned}$$

where $SW_{v}$is denoted as Eq. 14, and $W_{v,u}$ is denoted as Eq. 17.

$Wmatrix_i(v,u)$ describes the weight of protein interaction (*v*, *u*) in sub-network. Here, *i* is the count of the sub-networks, with $i \in [1, m]$. For Saccharomyces cerevisiae, $m=12$, and for Drosophila melanogaster, $m=34$. $Wmatrix_i(v,u)$ ranges from $[0,+1]$, which is denoted as follows:

19 $$\begin{aligned} Wmatrix_i(v,u) = GOW(v,u) \cdot Ap_{i}(v) \cdot Ap_{i}(u) \cdot PCW(v,u) \cdot SW_{v} \cdot SW_{u} \end{aligned}$$

where, *GOW*(*v*, *u*) is denoted as Eq. 7, $Ap_{i}(v)$ and $Ap_{i}(u)$ are all denoted as Eq. 6, *PCW*(*v*, *u*) is denoted as Eq. 10, $SW_{v}$ and $SW_{u}$ are all denoted as Eq. 14.

#### Seed expansion in sub-networks

The expansion process is performed simultaneously in all sub-networks and terminates when $|K |= n$. If $|K|< n$, we select a protein called $max\_Wmatrix\_node$ from $Nei\_K_i$, where $i\in [1,m]$. Owing to $Nei\_K_i \cap K = \emptyset$, the protein selected from $Nei\_K_i$ is not in *K*. For each protein (denoted as *Nei*) in $Nei\_K_i$, calculate $score\_W_{Nei}$ based on *Wmatrix*, $score\_W_{Nei}$ is defined as follows:

20 $$\begin{aligned} score\_W_{Nei} = \sum \limits _{v \in useNei}^{useNei}Wmatrix_{i}(Nei, v) \end{aligned}$$

where, $useNei = N_{Nei} \bigcap K$, $N_{Nei}$ is the set of neighbors of protein *Nei*. Among the neighbors of protein *Nei*, only those within *K* are taken into consideration. When protein *Nei* is more closely connected with set *useNei*, the value of $score\_W_{Nei}$ is higher. This approach allows us to effectively leverage the topological characteristics of predicted essential proteins within sub-networks.

The selection process of protein $max\_Wmatrix\_node$ consists of two steps. In the first step, we find the maximum $score\_W_{Nei}$ in each sub-network. For all $Nei \in Nei\_K_i$, the maximum $score\_W_{Nei}$ is denoted as $sub\_max\_Wmatrix_i$. In the second step, after calculating all the sub-networks, we gather all the $sub\_max\_Wmatrix_i$ values, where $i\in [1,m]$. The maximum value among these is denoted as $max\_Wmatrix$, and its corresponding node is $max\_Wmatrix\_node$.

We add the node $max\_Wmatrix\_node$ into *K* if it is not already in *K*, and if it exists in $V_i$, we also add it to $K_i$. If we change the node in *K*(by adding or deleting it), we should also change the corresponding node in $K_i$(by adding or deleting it). Among $Nei\_K_i$ of all the sub-networks, $max\_Wmatrix\_node$ is the node with the closest connections to the predicted essential proteins set *K* and possesses crucial biological characteristics.

#### Seed expansion method with error correction mechanism

The initialization of $K_i$ and *K* is random, and the expansion process is based on $Nei\_K_i$ of sub-networks. If the essentiality of the seed in $K_i$ is low, then there is a high probability that the essentiality of their neighboring nodes will also be low. In other words, the essentiality of the node selected from $Nei\_K_i$ will also be low. Therefore, we add an error correction mechanism to filter out the nodes with low essentiality in *K*. The error correction mechanism is based on $score\_initial$ calculates by Eq. 18, which describes the essentiality of protein in the whole PPI network.

The error correction mechanism consists of two main steps. In the first step, we find two nodes based on $score\_initial$. The first node is $min\_initial\_node$, which has minimum $score\_initial$(denoted as $min\_initial$) of all proteins in *K* that have not been removed. The second node is $max\_initial\_node$, which has maximum $score\_initial$(denoted as $max\_initial$) of all proteins in $Nei\_K$. Since $Nei\_K \cap K = \emptyset$, $max\_initial\_node$ is not in *K*. For all proteins in $Nei\_K$, $max\_initial\_node$ has the most important topological and biological characteristics. The selection of $min\_initial\_node$ and $max\_initial\_node$ is based on the whole PPI network. In the second step, we include one node in *K* while removing another node from *K*. If $max\_initial > min\_initial$, we add $max\_initial\_node$ to *K* if it is not already in *K*. Moreover, if $min\_initial\_node$ has not been removed from *K* before, we remove it. A node in *K* can only be removed one time. Subsequently we update $K_i$ and the state of $min\_initial\_node$ is altered to ’removed’. In order to ensure $|K |$ increases monotonically with the increase of the number of iterations, we remove $min\_initial\_node$ from *K* if and only if $max\_initial\_node$ has been added to *K* during this particular iteration.

The error correction mechanism is based on $score\_initial$, which is the weight based on biological data. The selection of $max\_initial\_node$ integrates the topological characteristics of predicted essential proteins in the whole PPI network.

### Analysis of biological data

We integrate protein complex, gene expression data, GO annotations and subcellular localization into the seed expansion process. More specifically, each biological characteristic is employed to initialize $score\_initial$ and *Wmatrix*. In order to analyze the effect of each biological characteristic on the final prediction results, we delete the weighting method based on biological data one by one. For example, in Eq. 18, let $GOW(v,u) = 1$. In other words, we no longer use GO annotations to weight *Wmatrix*, while everything else remains the same. Specifically, Eqs. 18 and 19 are redefined as Eqs. 21 and 22 as follows:

21 $$\begin{aligned} score\_initial_{v} = (\sum _{u \in N_{v}}^{N_{v}}GOW(v,u)^{\alpha _1} \cdot GW(v,u)^{\alpha _2} \cdot PCW(v,u)^{\alpha _3} ) \cdot SW_{v}^{\alpha _4} \end{aligned}$$

where $W_{v,u}$ of Eq. 17 is redefined as $GOW(v,u)^{\alpha _1} \cdot GW(v,u)^{\alpha _2} \cdot PCW(v,u)^{\alpha _3}$ and $SW_{v}$ of Eq. 14 is redefined as $SW_{v}^{\alpha _4}$.

22 $$\begin{aligned} Wmatrix_i(v,u) = GOW(v,u)^{\alpha _5} \cdot Ap_{i}(v,u)^{\alpha _6} \cdot PCW(v,u)^{\alpha _7} \cdot SW(v,u)^{\alpha _8} \end{aligned}$$

where $Ap_{i}(v,u) = Ap_{i}(v) \cdot Ap_{i}(u)$, $Ap_{i}(v)$ and $Ap_{i}(u)$ are denoted as Eq. 6, $SW(v,u) = SW_{v} \cdot SW_{u}$, $SW_{v}$ and $SW_{u}$ are denoted as Eq. 14. $\alpha _1$ to $\alpha _8$ determine which biological data will be deleted.

The value set $(\alpha _1,\alpha _2,...\alpha _8) = \{(0,1,...,1),(1,0,...,1),...,(1,1,...,0),(1,1,...,1)\}$ includes 9 groups of values, of which the ninth group consists entirely of 1 s. The values of the first eight groups are: $\alpha _1$ to $\alpha _8$ take 0 in sequence, while the remaining seven values are all 1 s. For example, the first group is (0, 1, 1, 1, 1, 1, 1, 1).

**Table 2:** The effect of biological data

Dataset	Measures	$score\_initial$				*Wmatrix*				All
		GO	Gene	Com	Sub	GO	Gene	Com	Sub	
DIP	SN	0.5467	0.5467	0.5510	0.5313	0.5441	0.5476	**0.5656**	0.5296	0.5476
	SP	0.8383	0.8383	0.8395	0.8337	0.8375	0.8385	**0.8439**	0.8332	0.8385
	PPV	0.5012	0.5012	0.5051	0.4870	0.4988	0.5020	**0.5185**	0.4855	0.5020
	NPV	0.8615	0.8615	0.8628	0.8568	0.8607	0.8618	**0.8673**	0.8563	0.8618
	F	0.5230	0.5230	0.5270	0.5082	0.5205	0.5238	**0.5410**	0.5066	0.5238
	ACC	0.7715	0.7715	0.7734	0.7644	0.7703	0.7718	**0.7801**	0.7636	0.7718
BioGRID	SN	0.5608	0.5641	0.5742	0.5575	0.5633	0.5633	**0.5967**	0.5575	0.5642
	SP	0.8340	0.8349	0.8376	0.8332	0.8347	0.8347	**0.8437**	0.8331	0.8350
	PPV	0.4773	0.4801	0.4887	0.4745	0.4794	0.4794	**0.5078**	0.4747	0.4801
	NPV	0.8754	0.8764	0.8792	0.8745	0.8761	0.8761	**0.8856**	0.8745	0.8764
	F	0.5157	0.5188	0.5280	0.5126	0.5180	0.5180	**0.5486**	0.5126	0.5188
	ACC	0.7759	0.7774	0.7816	0.7745	0.7770	0.7770	**0.7912**	0.7745	0.7774
Fruitfly	SN	0.2880	0.2880	0.2738	**0.2982**	0.2880	0.2819	0.2535	0.2941	0.2860
	SP	0.9128	0.9128	0.9118	**0.9134**	0.9128	0.9123	0.9104	0.9132	0.9126
	PPV	0.1825	0.1825	0.1735	**0.1889**	0.1825	0.1787	0.1607	0.1864	0.1812
	NPV	0.9499	0.9499	0.9489	**0.9506**	0.9499	0.9495	0.9475	0.9503	0.9498
	F	0.2234	0.2234	0.2124	**0.2313**	0.2234	0.2187	0.1967	0.2282	0.2219
	ACC	0.8732	0.8732	0.8714	**0.8745**	0.8732	0.8724	0.8688	0.8740	0.8729

**Fig. 4:**
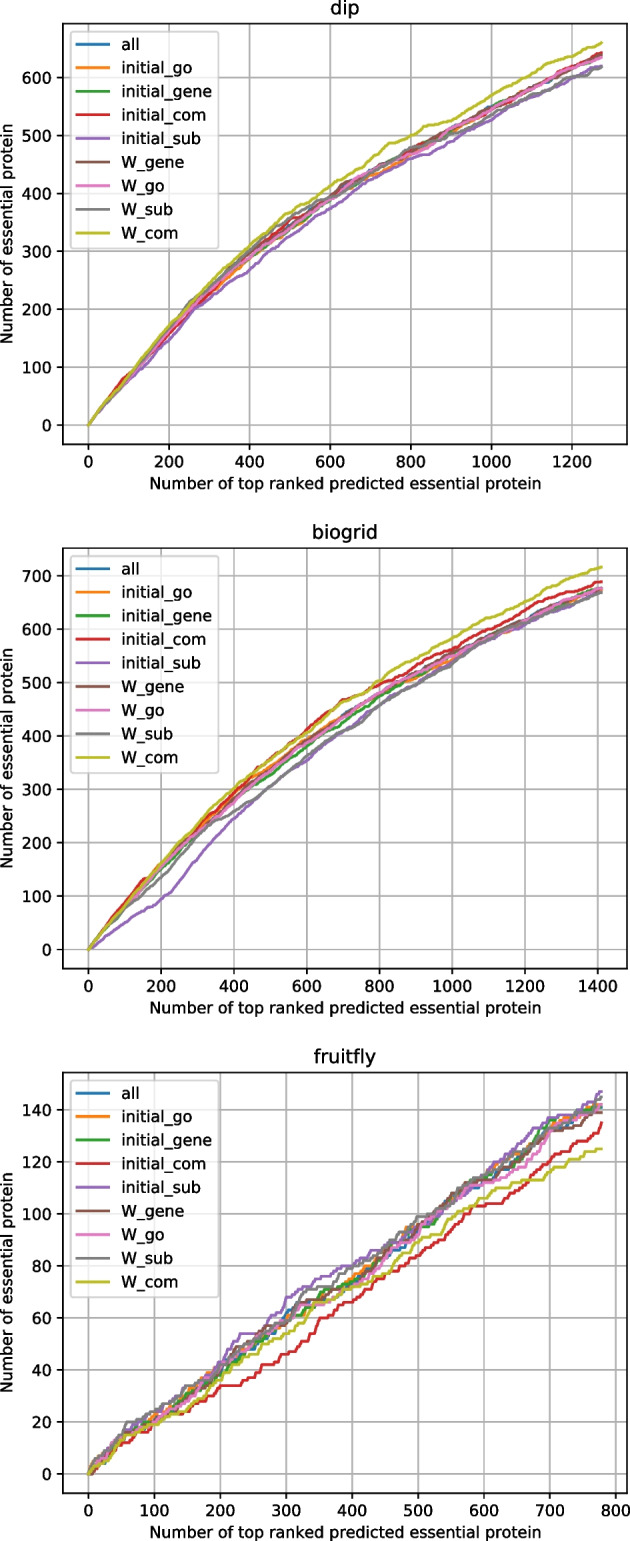
Jackknife curves of the effect of biological data

Table 2 compares the statistical measures of 9 methods. The 9 columns of Table 2 correspond to 9 groups of values in $(\alpha _1,\alpha _2,...\alpha _8)$. The defination of statistical measures can be found in the section ’Statistical measures’. Each column of statistical measures corresponds to the deletion of GO annotations, gene expression data, protein complex, and subcellular localization of the initialization of $score\_initial$ and *Wmatrix*, respectively. Figure 4 shows jackknife curves of the three aforementioned datasets. The labels $initial\_go$, $initial\_gene$, $initial\_com$, $initial\_sub$, $W\_go$, $W\_gene$, $W\_com$, $W\_sub$ and *all* correspond to the 9 groups of values in $(\alpha _1,\alpha _2,...\alpha _8)$. The statistical measures and jackknife curves achieved the same experimental results. For Saccharomyces cerevisiae, when initializing *Wmatrix* without protein complex, the DIP and BioGRID datasets achieve the best results. The value set $(\alpha _1,\alpha _2,...\alpha _8) = (1,1,1,1,1,1,0,1)$ corresponds to these optimal results, which we will employ in subsequent experiments. For Drosophila melanogaster, when initializing $score\_initial$ without subcellular localization, we achieve the best results, the value set $(\alpha _1,\alpha _2,...\alpha _8) = (1,1,1,0,1,1,1,1)$. We also employ these results in the follow-on experiments. The method with the best results is SESN.

## Experimental results and discussion

### Statistical measures

We compare the performance of our method with other identification methods by six statistical measures. These statistical measures can also be used to analyze the effect of each component and biological data on the final results. We define sensitivity (SN), specificity (SP), positive predictive value (PPV), negative predictive value (NPV), F-measure (F), and accuracy (ACC) as follows: $SN = \frac{TP}{TP+FN}$, $SP = \frac{TN}{TN+FP}$, $PPV = \frac{TP}{TP+FP}$, $NPV = \frac{TN}{TN+FN}$, $F = \frac{2 \cdot SN \cdot PPV}{SN+PPV}$, $ACC = \frac{TP+TN}{TP+FP+TN+FN}$. Where TP is true positives; FP is false positives; TN is true negatives; and FN is false positives. The larger these statistical measures, the higher the accuracy of the corresponding essential protein identification method.

### Jackknife curves

We plot jackknife curves to display the number of true positives for essential proteins in the predicted set of essential proteins as the ranking increases. We consider a protein’s ranking to be higher if it is added to the set *K* earlier.

### Analysis of each component

In order to validate the effectiveness of each component of SESN, we remove one or several components. Specifically, we remove the sub-networks component, the error correction mechanism, the seed expansion component, and the subcellular localization selection. When we remove the sub-networks component, the process of seed expansion is based on the whole network, and this method is named $rm\_sub$. We remove the error correction mechanism, the seed expansion is only based on *Wmatrix* with no error correction mechanism. This method is named $rm\_correction$. We remove the seed expansion component, and the process of essential protein identification is only based on *Wmatrix* or $score\_initial$. For *Wmatrix*, we initialize the weight of a protein based on the following equation: $score\_Wmatrix_{v} = \sum _{u \in N_{v}}^{N_{v}} Wmatrix_{v,u}$, where *Wmatrix* is denoted as Eq. 19. This method is named *Wmatrix*. The method only based on $score\_initial$ is named $score\_initial$, where the weight of the protein is based on Eq. 18. In section ’Subcellular localization’, we select some subcellular localizations highly correlated to essential proteins, to validate the effectiveness of this component, we use all subcellular localizations, this method is named $all\_subcellular$.

**Table 3:** Effectiveness of each part of SESN

Datasets	Measures	$rm\_sub$	$rm\_correction$	*Wmatrix*	$score\_initial$	$all\_subcellular$	**SESN**
DIP	SN	0.5493	0.5304	0.5501	0.5484	0.5467	**0.5656**
	SP	0.8390	0.8334	0.8393	0.8388	0.8382	**0.8439**
	PPV	0.5035	0.4863	0.5043	0.5027	0.5012	**0.5185**
	NPV	0.8623	0.8565	0.8626	0.8620	0.8615	**0.8673**
	F	0.5254	0.5074	0.5262	0.5246	0.5230	**0.5410**
	ACC	0.7726	0.7640	0.7730	0.7722	0.7715	**0.7801**
BioGRID	SN	0.5658	0.5542	0.5658	0.5650	0.5775	**0.5967**
	SP	0.8354	0.8322	0.8354	0.8351	0.8385	**0.8437**
	PPV	0.4816	0.4716	0.4816	0.4809	0.4915	**0.5078**
	NPV	0.8768	0.8735	0.8768	0.8766	0.8801	**0.8856**
	F	0.5203	0.5096	0.5203	0.5195	0.5310	**0.5486**
	ACC	0.7780	0.7730	0.7780	0.7777	0.7830	**0.7912**
Fruitfly	SN	0.2819	0.2799	0.2657	0.2779	0.2941	**0.2982**
	SP	0.9123	0.9122	0.9112	0.9121	0.9132	**0.9134**
	PPV	0.1787	0.1774	0.1684	0.1761	0.1864	**0.1889**
	NPV	0.9495	0.9493	0.9483	0.9491	0.9503	**0.9506**
	F	0.2187	0.2172	0.2061	0.2155	0.2282	**0.2313**
	ACC	0.8724	0.8722	0.8704	0.8719	0.8740	**0.8745**

**Fig. 5:**
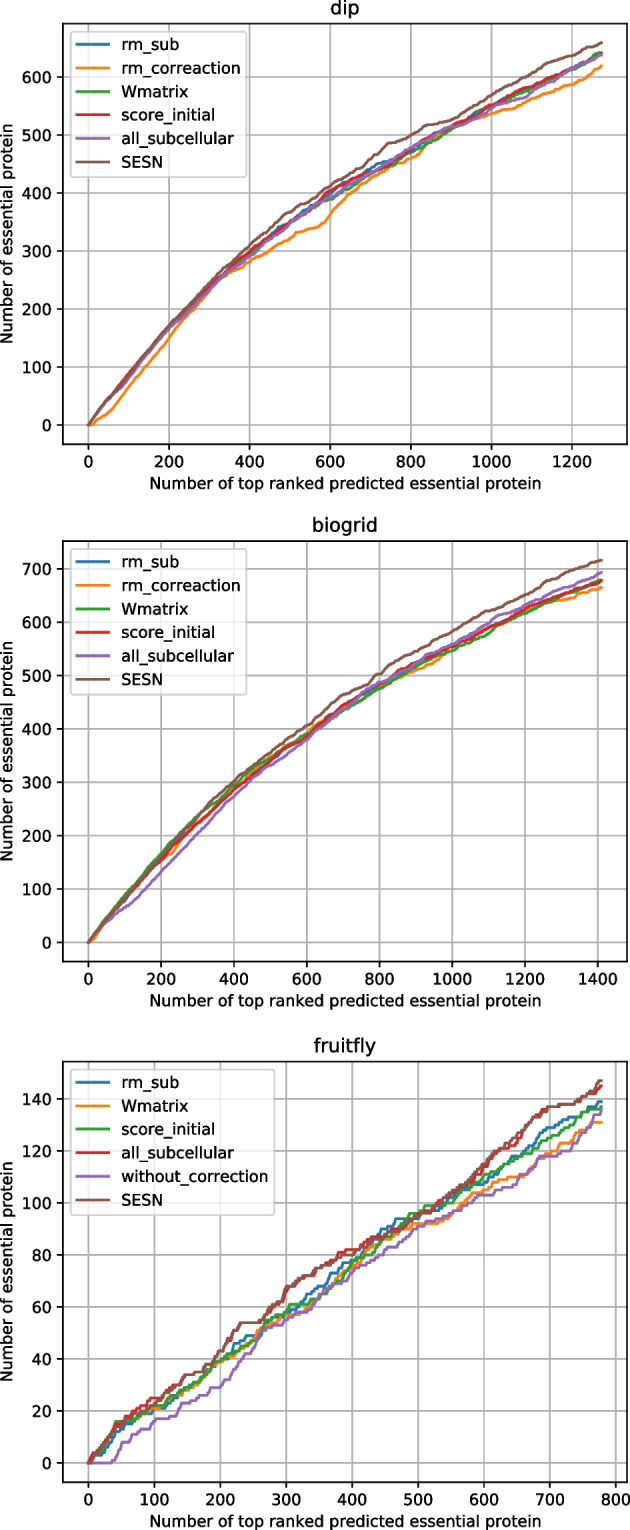
Jackknife curves of the effect of each component of SESN

As shown in Table 3 and Fig. 5, compared with $rm\_sub$, $rm\_correction$, *Wmatrix*, $score\_initial$, and $all\_subcellular$, SESN achieves the best results in three datasets. All components are effective to SESN. For DIP and BioGRID, all components show a significant gap with SESN, especially $rm\_correction$, which indicates that error correction mechanism is the most effective component. For Drosophila melanogaster, $without\_correction$ corresponds to $rm\_correction$ in Table 3. All components are effective except $all\_subcellular$. The statistic measures of SESN are slightly higher than those of $all\_subcellular$, and their jackknife curves basically coincide, which means that the selection of subcellular localization plays a small role in this case. It also demonstrates that the unselected subcellular localizations do not work in predicting essential proteins.

### Analysis of the performance of SESN and other methods

To validate the performance of SESN, we compare it with other methods: CPPK, CEPPK, RWEP, SIGEP, RWO and TS-PIN.

The algorithms CPPK and CEPPK are based on the whole protein-protein interaction data. To filter false positive interactions, SESN integrate multiple biological characteristics to construct weighted PPI sub-networks. The algorithms CPPK and CEPPK randomly select k ($k=100$) known essential proteins as prior knowledge and add them to a set *K*. However, the performance of CPPK and CEPPK excessively depends on the number of essential proteins. The algorithm SESN does not use essential proteins as prior knowledge. We randomly select a protein in each sub-network and add it to a set $K_i$. $K = \bigcup _{i=1}^m K_i$. Different from the CPPK and CEPPK algorithms, *K* is the set of predicted essential proteins, not known essential proteins. When the CPPK and CEPPK algorithms perform node expansion on set *K* within the whole PPI network, they select the neighbor node of *K* with the highest score and add it to set *K*. Due to the fact that the node expansion process of the CPPK and CEPPK algorithms is based on the neighbor nodes of *K*, it results in considering only the topological characteristics of set *K* within the whole PPI network. The algorithm SESN considers topological characteristics of predicted essential proteins in $K_i$ and *K*. The seed expansion process of algorithm SESN is based on the neighbor nodes of $K_i$, which integrates the topological characteristics of predicted essential proteins in the sub-networks. The error correction mechanism of algorithm SESN is based on the neighbor nodes of *K*, which integrates the topological characteristics of predicted essential proteins in the whole PPI network. CPPK and CEPPK are only applied to Saccharomyces cerevisiae, they randomly select 100 essential proteins as prior knowledge. For Drosophila melanogaster, the fruitfly dataset only has 493 essential protein, we randomly select 20 essential proteins as prior knowledge. The same as SESN, we regard the earlier a protein is selected as a predicted essential protein, the higher its score. SESN does not use essential proteins as prior knowledge, but SESN detects essential proteins more effectively than CPPK and CEPPK.

RWEP integrates the same biological properties as SESN. As shown in Table 5 and Fig. 7, to achieve optimal results, parameter $\lambda$ of RWEP is set to 0.2, 0.1, and 0.9 for DIP, BioGRID, and fruitfly datasets, respectively. SESN analyzes the effect of each biological data on the final prediction results, and adopts the corresponding biological data with the best results. The experimental results show that SESN outperforms RWEP significantly, demonstrating the effectiveness of analyzing biological data from a different perspective.

SIGEP presents a p-value calculation method in which both degree and local clustering coefficient are used as test statistic. Proteins are sorted according to p-values. SIGEP does not integrate any biological data, and its performance is inferior to that of SESN, which indicates that the integration of biological data improves the performance of SESN.

RWO uses orthologous relationships to connect yeast and human PPI. Since RWO does not give the orthologous relationships applied to the fruitfly, we compare RWO and SESN on yeast datasets: DIP and BioGRID. SESN does not integrate orthologous relationships, but its performance is significantly better than RWO.

**Table 4:** Comparison of statistical measures between SESN and other methods

Datasets	Measures	*CPPK*	*CEPPK*	*RWEP*	*SIGEP*	*RWO*	**SESN**
DIP	SN	0.4893	0.5056	0.5347	0.4627	0.4327	**0.5656**
	SP	0.8212	0.8260	0.8347	0.8133	0.8044	**0.8439**
	PPV	0.4485	0.4635	0.4902	0.4242	0.3967	**0.5185**
	NPV	0.8440	0.8490	0.8579	0.8359	0.8267	**0.8673**
	F	0.4680	0.4836	0.5115	0.4426	0.4139	**0.5410**
	ACC	0.7451	0.7526	0.7660	0.7330	0.7192	**0.7801**
BioGRID	SN	0.5383	0.5367	0.5675	0.5367	0.4933	**0.5967**
	SP	0.8280	0.8276	0.8359	0.8276	0.8158	**0.8437**
	PPV	0.4582	0.4567	0.4830	0.4567	0.4199	**0.5078**
	NPV	0.8691	0.8686	0.8774	0.8686	0.8563	**0.8856**
	F	0.4950	0.4935	0.5218	0.4935	0.4536	**0.5486**
	ACC	0.7664	0.7657	0.7788	0.7657	0.7473	**0.7912**
Fruitfly	SN	0.2190	0.2130	0.2698	0.1866		**0.2982**
	SP	0.9081	0.9077	0.9115	0.9059		**0.9134**
	PPV	0.1388	0.1350	0.1710	0.1183		**0.1889**
	NPV	0.9450	0.9446	0.9486	0.9428		**0.9506**
	F	0.1699	0.1652	0.2093	0.1448		**0.2313**
	ACC	0.8644	0.8637	0.8709	0.8603		**0.8745**

**Fig. 6:**
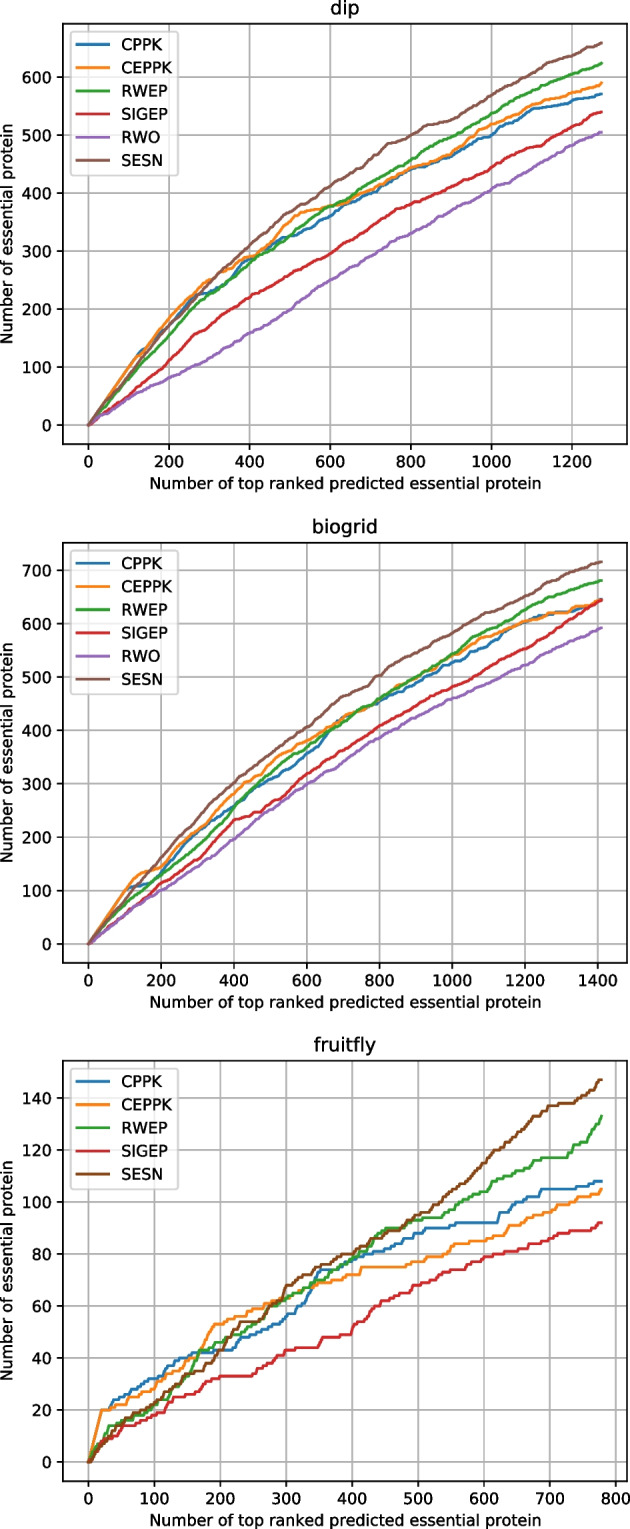
Jackknife curves of SESN and other methods

The statistical measures of these methods are shown in Table 4. In the three datasets, the values of six statistical measures of SESN are the highest among all these methods. Jackknife curves of these methods are shown in Fig. 6, revealing that SESN significantly better than other methods.

TS-PIN is an algorithm aimed at optimizing PPI networks. TS-PIN refines the PPI network by removing edges in it. The initial PPI network is denoted as PPI. By utilizing TS-PIN, specific edges are removed from the PPI network to generate the refined network, which is designated as TSPPI. To assess the effectiveness of TS-PIN for SESN, we feed the TSPPI network into SESN and execute all the SESN steps. This combined approach is denoted as TS-PIN-SESN. The distinction between TS-PIN-SESN and SESN lies solely in the input network. The TSPPI network employed by TS-PIN-SESN is a subset of the PPI network used by SESN, resulting in the two algorithms utilizing distinct datasets. To ensure the validity of the comparative experimental outcomes, we implemented the subsequent two procedures on the output results of the two comparative algorithms. 1: For the output results of the SESN algorithm, only the proteins appearing in the TSPPI network were retained. In this manner, the experimental results of TS-PIN-SESN and SESN are based on the TSPPI network. The statistical measures and Jackknife curves of SESN and TS-PIN-SESN on TSPPI network are shown in Table 6 and Fig. 8. The experimental results show that the TS-PIN-SESN algorithm do not improve the identification accuracy of essential proteins in the TSPPI network. Both TS-PIN-SESN and SESN yield identical experimental results for the BioGRID and fruitfly datasets. However, for the experimental results regarding the DIP dataset, TS-PIN-SESN exhibits even poorer performance. 2: We assign a score of 0 to proteins that have been removed from the PPI network, and subsequently add these proteins along with their scores to the output results of the TS-PIN-SESN algorithm. In this manner, the experimental results of both TS-PIN-SESN and SESN are based on the PPI network. The statistical measures and Jackknife curves of SESN and TS-PIN-SESN on PPI network are shown in Table 7 and Fig. 9. The experimental results show that the SESN algorithm performs better than TS-PIN-SESN. In summary, the TS-PIN algorithm is ineffective for SESN.

**Table 5:** Statistical measures of different values of $\lambda$ for RWEP

Datasets	Measures	0.1	0.2	0.3	0.4	0.5	0.6	0.7	0.8	0.9	1
DIP	SN	0.5004	0.5347	0.5338	0.5296	0.5201	0.5167	0.5047	0.5021	0.4996	0.5004
	SP	0.8245	0.8347	0.8344	0.8332	0.8304	0.8293	0.8258	0.8250	0.8242	0.8245
	PPV	0.4588	0.4902	0.4894	0.4855	0.4768	0.4737	0.4627	0.4603	0.4580	0.4588
	NPV	0.8474	0.8579	0.8576	0.8563	0.8534	0.8524	0.8487	0.8479	0.8471	0.8474
	F	0.4951	0.5115	0.5107	0.5066	0.4975	0.4943	0.4828	0.4803	0.4779	0.4786
	ACC	0.7581	0.7660	0.7656	0.7636	0.7593	0.7577	0.7522	0.7510	0.7499	0.7502
BioGRID	SN	0.5675	0.5667	0.5550	0.5475	0.5392	0.5308	0.5233	0.5200	0.5150	0.5133
	SP	0.8359	0.8356	0.8324	0.8304	0.8282	0.8259	0.8239	0.8230	0.8216	0.8212
	PPV	0.4830	0.4823	0.4723	0.4660	0.4589	0.4518	0.4454	0.4426	0.4383	0.4369
	NPV	0.8774	0.8771	0.8738	0.8716	0.8693	0.8669	0.8648	0.8638	0.8624	0.8619
	F	0.5218	0.5211	0.5103	0.5034	0.4958	0.4881	0.4812	0.4782	0.4736	0.4720
	ACC	0.7788	0.7784	0.7734	0.7702	0.7667	0.7631	0.7599	0.7585	0.7564	0.7557
Fruitfly	SN	0.2535	0.2556	0.2637	0.2657	0.2657	0.2677	0.2677	0.2677	0.2698	0.2535
	SP	0.9104	0.9106	0.9111	0.9112	0.9112	0.9114	0.9114	0.9114	0.9115	0.9104
	PPV	0.1607	0.1620	0.1671	0.1684	0.1684	0.1697	0.1697	0.1697	0.1710	0.1607
	NPV	0.9475	0.9476	0.9482	0.9483	0.9483	0.9485	0.9485	0.9485	0.9486	0.9475
	F	0.1967	0.1983	0.2046	0.2061	0.2061	0.2077	0.2077	0.2077	0.2093	0.1967
	ACC	0.8688	0.8691	0.8701	0.8704	0.8704	0.8706	0.8706	0.8706	0.8709	0.8688

**Fig. 7:**
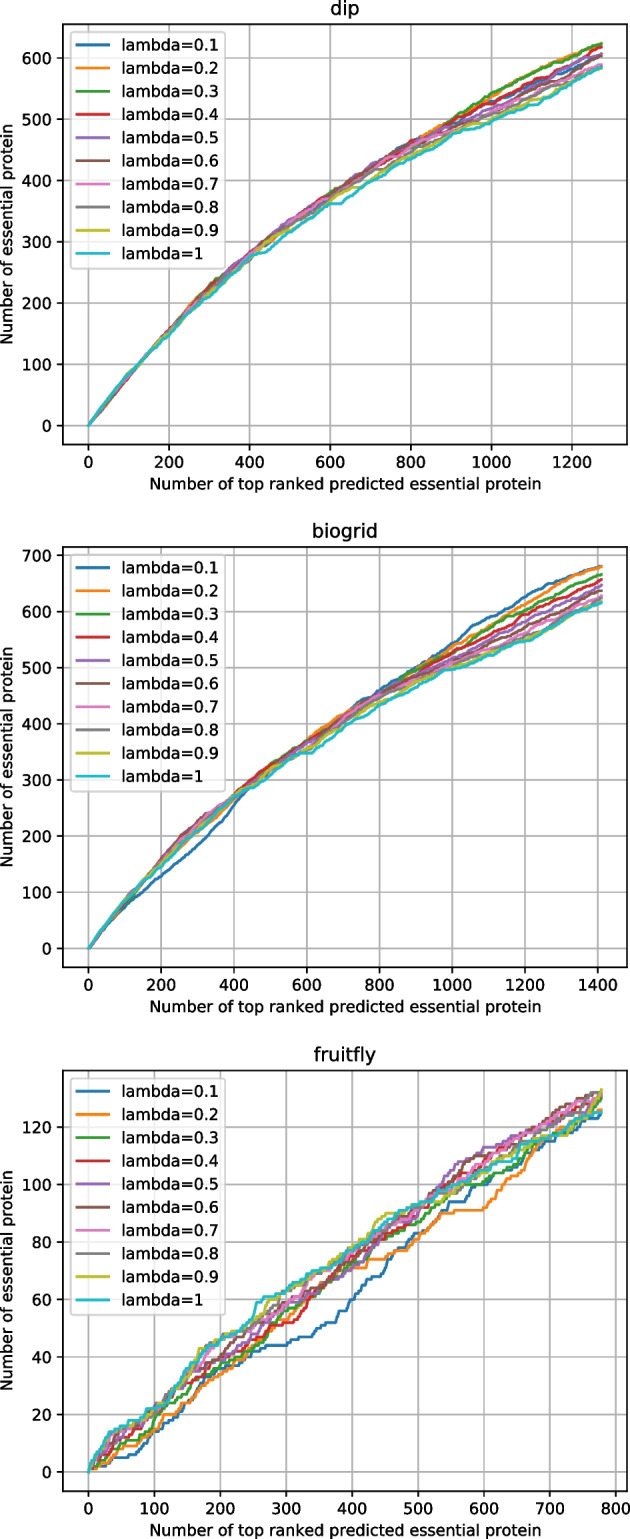
Jackknife curves of the effect of parameter $\lambda$ of RWEP

**Table 6:** Comparison of statistical measures between SESN and TS-PIN-SESN on TSPPI network

Datasets	Measures	TS-PIN-SESN	**SESN**
DIP	SN	0.4847	**0.4872**
	SP	0.8742	**0.8755**
	PPV	0.6708	**0.6743**
	NPV	0.7622	**0.7634**
	F	0.5627	**0.5657**
	ACC	0.7394	**0.7412**
BioGRID	SN	0.5186	**0.5186**
	SP	0.8743	**0.8743**
	PPV	0.6558	**0.6558**
	NPV	0.7973	**0.7973**
	F	0.5791	**0.5791**
	ACC	0.7619	**0.7619**
Fruitfly	SN	0.1618	**0.1618**
	SP	0.9090	**0.9090**
	PPV	0.2	**0.2**
	NPV	0.8852	**0.8852**
	F	0.1789	**0.1789**
	ACC	0.8169	**0.8169**

**Table 7:** Comparison of statistical measures between SESN and TS-PIN-SESN on PPI network

Datasets	Measures	TS-PIN-SESN	**SESN**
DIP	SN	0.4696	**0.5656**
	SP	0.8153	**0.8439**
	PPV	0.4305	**0.5185**
	NPV	0.8380	**0.8673**
	F	0.4492	**0.5410**
	ACC	0.7361	**0.7801**
BioGRID	SN	0.5125	**0.5967**
	SP	0.8210	**0.8437**
	PPV	0.4362	**0.5078**
	NPV	0.8618	**0.8856**
	F	0.4713	**0.5486**
	ACC	0.7554	**0.7912**
Fruitfly	SN	0.2252	**0.2982**
	SP	0.9085	**0.9134**
	PPV	0.1427	**0.1889**
	NPV	0.9455	**0.9506**
	F	0.1747	**0.2313**
	ACC	0.8652	**0.8745**

**Fig. 8:**
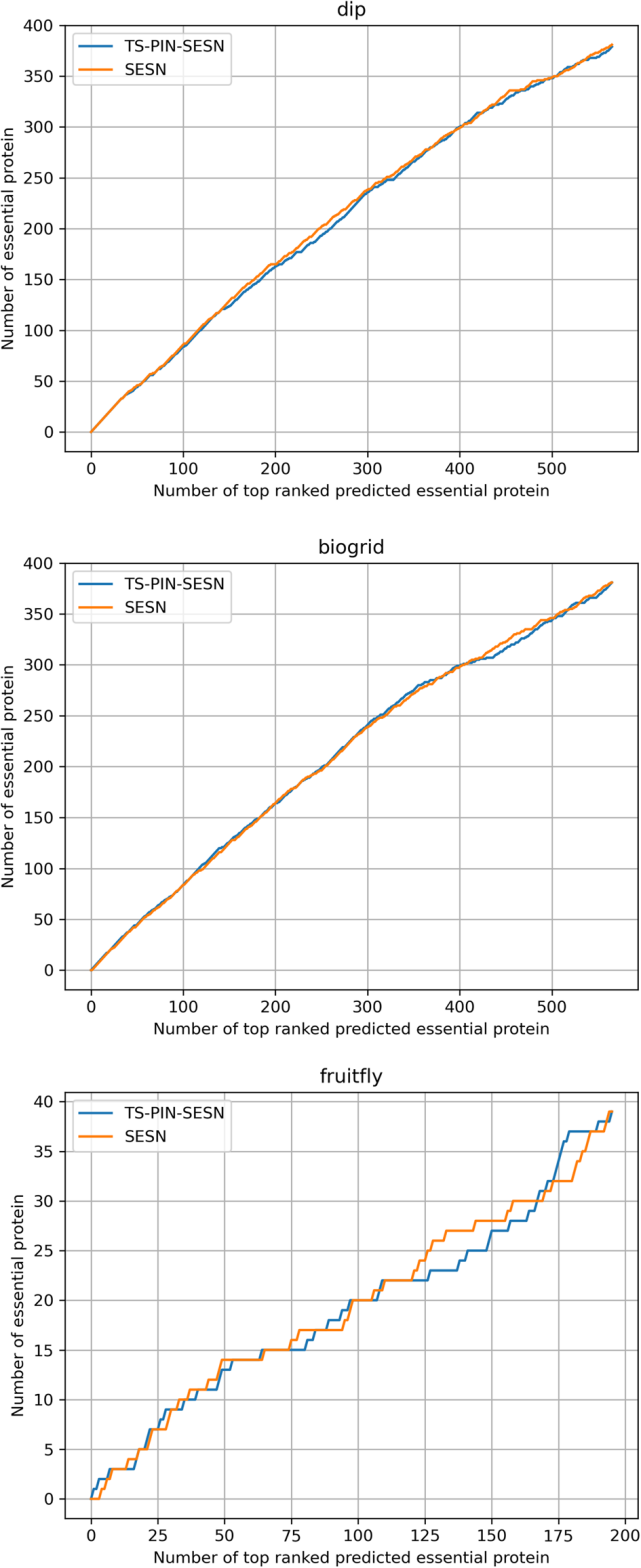
Jackknife curves of SESN and TS-PIN-SESN on TSPPI network

**Fig. 9:**
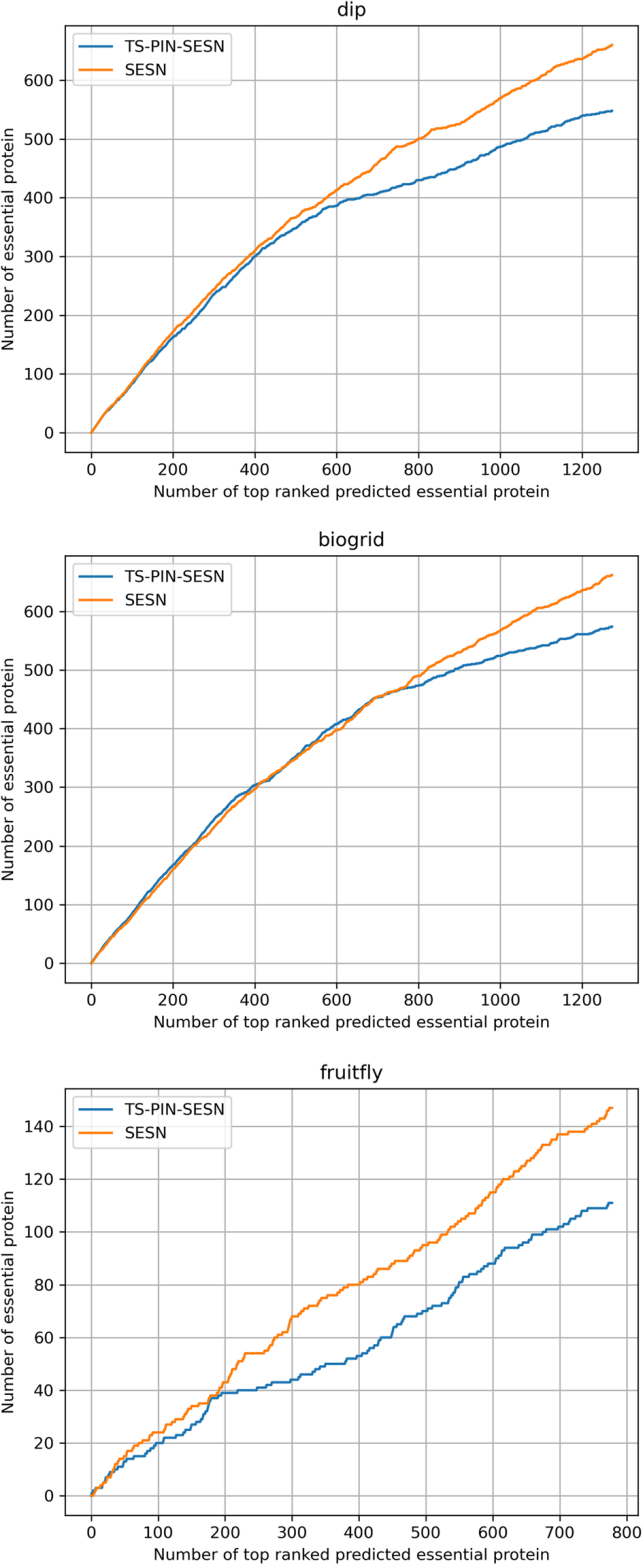
Jackknife curves of SESN and TS-PIN-SESN on PPI network

## Conclusions

Essential proteins are crucial for maintaining vital biological functions. Identifying essential proteins is of great significance for biology and pathology. In recent years, a large number of algorithms based on protein-protein interaction (PPI) networks have been proposed to identify essential proteins. However, PPI data obtained through high-throughput technology often contain many false positives. This will seriously affect the accuracy of identifying essential proteins. Therefore, further research is needed to improve the accuracy of essential protein identification.

In this paper, we propose a novel method named SESN for identifying essential proteins. SESN is a seed expansion method based on protein-protein interaction (PPI) sub-networks and biological characteristics. To filter out false positive interactions in PPI networks, SESN constructs PPI sub-networks using gene expression data. Seed expansion is performed simultaneously in each sub-network, where each sub-network randomly selects a protein as a seed, and the expansion results are summarized for the entire PPI network. The error correction mechanism filters out low-essentiality proteins that have been expanded. SESN adopts the biological data combination with the best experimental results. The output of SESN is a set of predicted essential proteins.

The analysis of each component of SESN shows that all components are effective, especially the error correction mechanism. The comparison experiments are conducted on three datasets of two species(DIP, BioGRID, fruitfly). Experiment results show that compared with other methods(CPPK, CEPPK, RWEP, SIGEP, RWO, and TS-PIN), SESN achieves the best results in three datasets. SESN may provide a useful tool for future research on prediction of essential proteins.

## Data Availability

The processed dataset and source codes are available in https://github.com/zhaohe555/SESN.git

## Abbreviations

PPI: Protein–protein interaction
GO: Gene ontology
SESN: A seed expansion method based on PPI sub-networks and multiple biological data to identify essential proteins
